# Supplementary material for: The interactome of intact mitochondria by cross-linking mass spectrometry provides evidence for coexisting respiratory supercomplexes
Source: Mol Cell Proteomics. 2017 Dec 8;17(2):216–32. doi: 10.1074/mcp.RA117.000470 (PMC5795388; doi:10.1074/mcp.RA117.000470)
Supplement: Supplemental Data [file supp_RA117.000470_133922_0_supp_23978_fzffwf.zip › spectra_annotation/mito_DR_spectra_annotation/217-1-23-1-19-1.pdf]

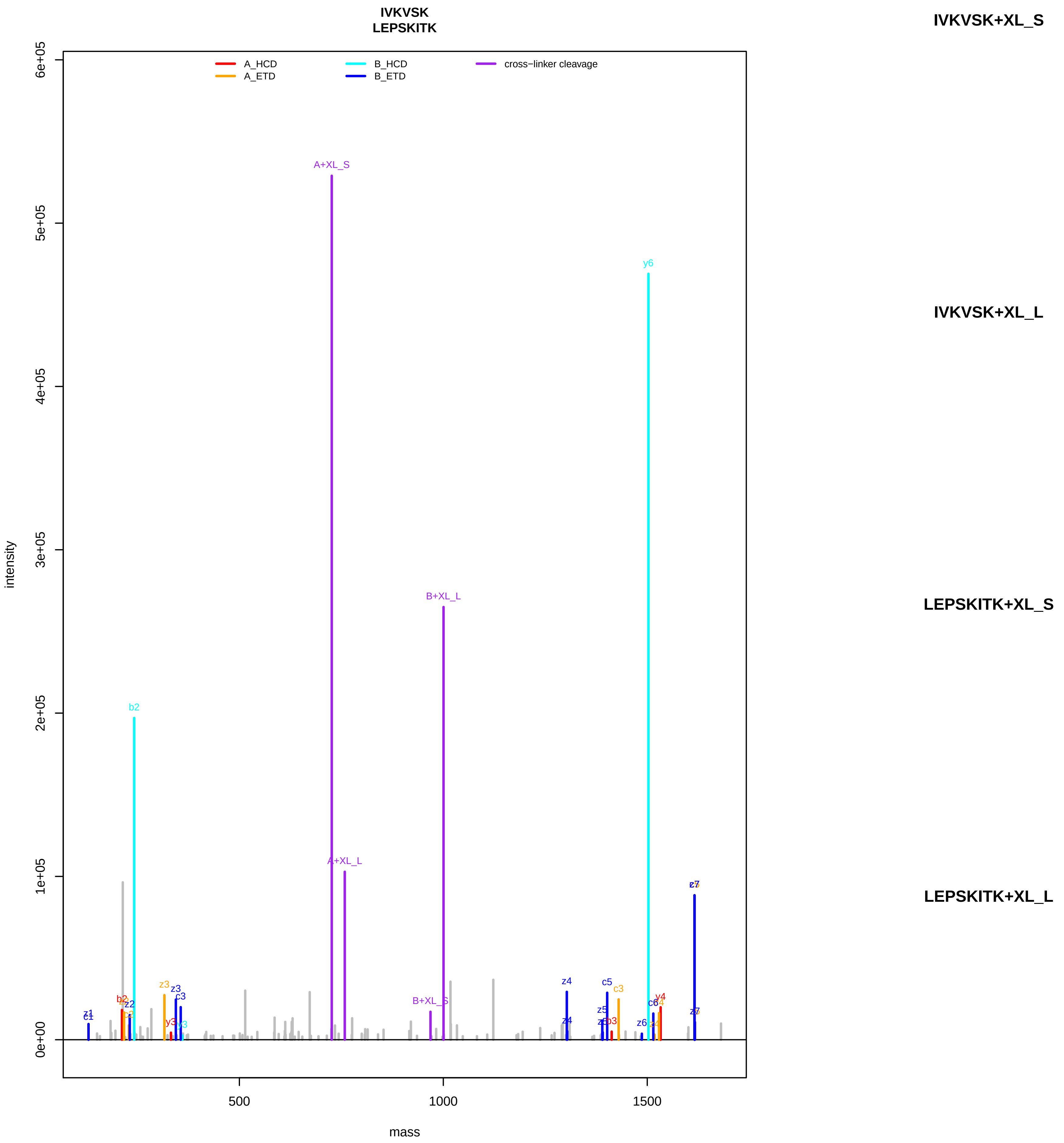
IVKVSK
LEPSKITK
A_HCD
A_ETD
B_HCD
B_ETD
cross−linker cleavage
A+XL_S
y6
B+XL_L
b2
A+XL_L
c7
z4
c5
c3
y4
c6
B+XL_S
z3
c3
z2
b2
z1
c1
y3
z5
b3
z6
z7
intensity
mass
0e+00
1e+05
2e+05
3e+05
4e+05
5e+05
6e+05
500
1000
1500
IVKVSK+XL_S
IVKVSK+XL_L
LEPSKITK+XL_S
LEPSKITK+XL_L
